# Is Vitamin D Deficiency Related to Increased Cancer Risk in Patients with Type 2 Diabetes Mellitus?

**DOI:** 10.3390/ijms22126444

**Published:** 2021-06-16

**Authors:** Anna Gabryanczyk, Sylwia Klimczak, Izabela Szymczak-Pajor, Agnieszka Śliwińska

**Affiliations:** 1 Department of Nucleic Acid Biochemistry, Medical University of Lodz, 251 Pomorska Str., 92-213 Lodz, Poland; anna.gabryanczyk@umed.lodz.pl (A.G.); izabela.szymczak@umed.lodz.pl (I.S.-P.); 2 Student Scientific Society of Civilization Diseases, Medical University of Lodz, 251 Pomorska, 92-213 Lodz, Poland; sylwia.cichuta@stud.umed.lodz.pl

**Keywords:** vitamin D deficiency, type 2 diabetes (T2DM), cancer

## Abstract

There is mounting evidence that type 2 diabetes mellitus (T2DM) is related with increased risk for the development of cancer. Apart from shared common risk factors typical for both diseases, diabetes driven factors including hyperinsulinemia, insulin resistance, hyperglycemia and low grade chronic inflammation are of great importance. Recently, vitamin D deficiency was reported to be associated with the pathogenesis of numerous diseases, including T2DM and cancer. However, little is known whether vitamin D deficiency may be responsible for elevated cancer risk development in T2DM patients. Therefore, the aim of the current review is to identify the molecular mechanisms by which vitamin D deficiency may contribute to cancer development in T2DM patients. Vitamin D via alleviation of insulin resistance, hyperglycemia, oxidative stress and inflammation reduces diabetes driven cancer risk factors. Moreover, vitamin D strengthens the DNA repair process, and regulates apoptosis and autophagy of cancer cells as well as signaling pathways involved in tumorigenesis i.e., tumor growth factor β (TGFβ), insulin-like growth factor (IGF) and Wnt-β-Cathenin. It should also be underlined that many types of cancer cells present alterations in vitamin D metabolism and action as a result of Vitamin D Receptor (VDR) and CYP27B1 expression dysregulation. Although, numerous studies revealed that adequate vitamin D concentration prevents or delays T2DM and cancer development, little is known how the vitamin affects cancer risk among T2DM patients. There is a pressing need for randomized clinical trials to clarify whether vitamin D deficiency may be a factor responsible for increased risk of cancer in T2DM patients, and whether the use of the vitamin by patients with diabetes and cancer may improve cancer prognosis and metabolic control of diabetes.

## 1. Introduction

Epidemiological studies reveal that morbidity of both diabetes and cancer is growing rapidly worldwide. According to the WHO, in 2014, there were 422 million people suffering from diabetes in the world, and in 2016, 1.6 million people died due to diabetes. It is estimated that in 2030 the number of diabetics will increase to 578 million worldwide [1]. In case of cancer incidence, the WHO reported 14 million patients in 2012, and estimated that there will be approximately 22 million of cases in 2032 [2].

Among different types of diabetes, the most common are type 1 and type 2, of which type 1 diabetes mellitus (T1DM) accounts for about 5% to 10% of all diabetes cases. Several studies have demonstrated that both T1DM and type 2 diabates (T2DM) are connected with increased cancer risk, however it seems that epidemiologically and biologically T2DM has a stronger link with cancer [3]. Due to the fact that cancer and T2DM share common risk factors, such as obesity, gender, smoking, and aging, it is believed that T2DM has been closely associated with many types of cancer, including cancers of the pancreas, liver, colorectal, breast, endometrium, and bladder [4]. It should be underlined that more men than women have diabetes, cancer and vitamin D deficiency. Although, the mechanisms for the association of T2DM and the incidence of cancer are not completely solved and understood, the insulin resistance/hyperinsulinism, hyperglycemia, oxidative stress and low grade chronic inflammation are biological factors related with T2DM that are considered as triggering factors of carcinogenesis [5,6,7,8]. As indicated by Habib and Rojna, the coexistence of diabetes and cancer is especially visible in people over 65. Namely, in this age group 26.9% subjects have diabetes and 60% have cancer, but 8–18% have both diseases [6].

The major feature of T2DM is insulin resistance, a defective response to physiological or increased endogenous or exogenous insulin concentration, that leads to hyperglycemia and hyperinsulinism. Insulin, not only governs metabolism, but also acts as a growth factor. Thus, hyperinsulinism stimulates abnormal activation of multiple cellular signaling cascades, strengthening growth factor-dependent cell proliferation. It is widely known that insulin elevates the bioavailability of insulin-like growth factor I (IGF-I) by promoting hepatic IGF-I synthesis and by decreasing hepatic protein secretion of the insulin like growth factor binding proteins 1 (IGFBP-1) and 2 (IGFBP-2) [9,10]. Thus, insulin can directly stimulate tumor growth, but numerous effects of its mitogenic and antiapoptotic action are driven by the IGF-I system, as observed in patients with high levels of circulating IGF-I with increased risk of developing several types of cancers, including prostate and breast [3,11]. Moreover, hyperinsulinemia/insulin resistance is a major factor responsible for increased occurrence of sex-hormone-related cancers via downregulation of sex-hormone-binding globulin (SHBG) leading to elevated estrogen and testosterone bioavailability [12].

It is well recognized that hyperglycemia-induced oxidative stress exerts harmful effects through dysregulation of multiple metabolic and molecular mechanisms affecting DNA, RNA, lipids and protein [13,14,15]. Of special importance are DNA damage, disturbances in DNA repair, mutation accumulation, activation of various pathways via lncRNA/miRNA, and posttranslational modifications of proteins that eventually lead to cell adaptations, which if sustained favor or drive towards malignant transformation [16,17,18].

Both chronic hyperglycemia and insulin resistance trigger to low grade chronic inflammation. This condition is characterized by the overproduction of numerous molecules, including adipose tissue released free fatty acids, interleukin-6 (IL-6), plasminogen activator inhibitor-1, leptin, tumor necrosis factor alpha (TNF-α) and monocyte chemoattractant protein (MCP-1). These overproduced molecules create a microenvironment promoting malignant transformation and cancer progression [4].

Vitamin D, a secosteroid, occurs in two major forms, vitamin D_2_ (ergocalciferol) and vitamin D_3_ (cholecalciferol). Vitamin D_2_ is synthesized by plants, fungi, yeasts from ergosterol by UVB, while vitamin D_3_ is produced in the skin by ultraviolet B radiation (UVB, 290–320 nm) and transformed from 7-dehydrocholesterol to cholecalciferol or supplied from food [19]. In the last few decades with the discovery of vitamin D receptors (VDR) in various tissues, the extra-skeletal effects of vitamin D became apparent and its biological functions are increasingly recognized. Vitamin D was demonstrated to regulate the cellular proliferation, differentiation, and immune modulation. And hence, apart from well recognized anti-rachitic action of vitamin D, multiple preclinical and clinical studies report its protection against several diseases, such as cancer, diabetes, hypertension, cardiovascular, autoimmune and dermatological diseases [20]. Although, the results of clinical trials are not conclusive, growing body of evidence reports vitamin D deficiency as a worldwide health problem that is related with the development of numerous diseases, including diabetes and cancer [8]. Recent findings reveal that the molecular mechanism of vitamin D involves insulin secretion and signaling, and thereby supports glycemic control and insulin sensitivity [21]. In light of the complex functions of vitamin D, covering the regulation of gene expression engaged in proliferation, differentiation, autophagy, apoptosis, epithelial–mesenchymal transition (EMT), modulation of cell–microenvironment interactions, antioxidants, enzymes, angiogenesis, and inflammation, it is suggested that vitamin D deficiency may be a key player of T2DM-cancer association, since it contributes to the exacerbation of disorders accompanying diabetes [21,22,23,24,25,26].

Therefore, in this review, we comprehensively show evidence for the pros and cons of the relationship between vitamin D deficiency and increased risk of cancer in patients with T2DM.

## 2. Methods

In order to compile the information on the connection between vitamin D deficiency and risk of cancer in T2DM patients, PUBMED, Google Scholar and PubChem databases were searched. The following articles types, in vitro and in vivo studies, observational and interventional clinical trials, and reviews published between 1990 and 2021 were used. The following keywords were utilized: vitamin D, VDR, CYP11A1, type 2 diabetes, vitamin D and cancer, vitamin D and diabetes, vitamin D and insulin resistance, vitamin D deficiency, molecular mechanism of vitamin D, vitamin D metabolism and cancer; vitamin D and anticancer activity, vitamin D and antioxidant properties, vitamin D and DNA repair, vitamin D and cell cycle, vitamin D and cell differentiation, vitamin D and apoptosis, vitamin D and proliferation, vitamin D and autophagy, vitamin D and angiogenesis in cancer, vitamin D deficiency and diabetes, vitamin D deficiency and diabetes and cancer.

## 3. Briefly about Vitamin D

### 3.1. The Classical Pathway of Vitamin D Metabolism

Both vitamin D_2_ and D_3_ bind to vitamin D-binding protein (VDBP), the complex formed is transported to the liver, where vitamin D is metabolized by the vitamin D 25-hydroxylase complex (CYP27A1 and CYP2R1) to form 25-hydroxycholecalciferol D (25(OH)D, calcidiol). The latter one is the main form of the vitamin found in the serum and its concentration is measured to determine in the actual body content of vitamin D. Thereafter, 25(OH)D 1α-hydroxylase (CYP27B1) metabolizes 25(OH)D to 1,25-dihydroxycholecalciferol D(1,25(OH)_2_D_3_, calcitriol) and/or 24,25-dihydroxycholecalciferol. This process takes place in the proximal tubules of kidneys and bone tissues as well as in other organs like lungs, skin, placenta, prostate, parathyroid glands and cells, including, dendritic cells, macrophages, and cancer cells. The most biologically active form of vitamin D is 1,25-dihydroxycholecalciferol. In turn, the function of 24,25-dihydroxycholecalciferol synthesized by vitamin D 24-hydroxylase (CYP24) is not fully understood. The resulting 1,25-dihydroxycholecalciferol D enters the circulatory system, where it binds to VDBP and in this form reaches target tissues and organs. When adequate levels of calcidiol and calcitriol controlled by 25(OH)D 24-hydroxylase (CYP24A1) are reached, this enzyme inactivates both by catalyzing hydroxylation at C-24 and C-23, forming biologically inactive calcitroic acid, which is excreted from the body with the bile [27,28].

### 3.2. The Alternative Pathway of Vitamin D Metabolism

**Figure 1:**
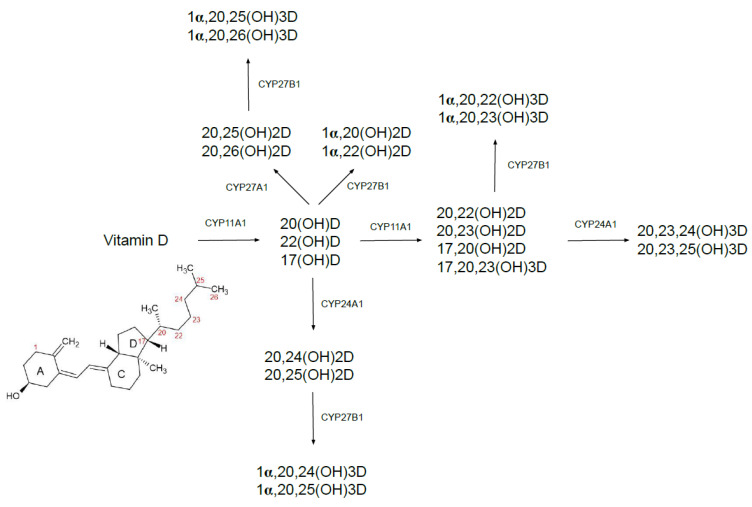
The metabolites obtained from the CYP11A1 mediated alternative vitamin D metabolic pathway [27,29,30,32].

The CYP11A1 pathway presented in Figure 1 is known as an alternative metabolic pathway of vitamin D. It was found that CYP11A1, that hydroxylates the side chains of vitamins D_2_ and D_3_ [29], catalyzes many coupled reactions with the synthesis of cholesterol, steroids and drug metabolism. Thus, through the CYP11A1 pathway, vitamin D may act as an alternative substrate for cholesterol synthesis. CYP11A1 hydroxylates vitamin D to 20(OH)D, 22(OH)D and 17(OH)D, subsequently, the next hydroxylation occurs and finally forms 20,23(OH)_2_D, 20,22(OH)_2_D, 17,20(OH)_2_D, and 17,20,23(OH)_3_D. The major product of the CYP11A1 pathway is 20(OH)D, which is used as a substrate by CYP27A1 (performs hydroxylation at C25 or C26) and CYP24A1 (performs hydroxylation at C24 or C25). The resulting products can be further hydroxylated by CYP27B1 at C-1α. It is established that the alternative pathway generates more than 21 hydroxymetabolites of vitamin D. It was found that 20(OH)D and its hydroxymetabolites are more effective in the inhibition of proliferation and inflammation as well as induction of differentiation, than calcitriol in the skin cells. The hydroxymetabolites intensify defense mechanisms against DNA damage and oxidative stress caused by UV radiation [27,29]. The CYP11A1-produced vitamin D metabolites, including 20(OH)D_3_, 20(OH)D_2_, 1,20(OH)_2_D_3_, and 20,23(OH)_2_D_3_, have also been demonstrated to suppress cell proliferation and stimulate cell differentiation via VDR [30,31]. 

CYP11A1 is expressed in the ovaries, testes, adrenal cortex and placenta, lungs, brain, gastrointestinal tract, immune cells, breasts, bones, prostate, and some cancer cells. It should be noted that the expression of CYP11A1 is at a low level and has little systemic effect. Interestingly, it was found that the CYP11A1-derived secosteroids inhibited melanocyte proliferation and were capable of preventing melanoma [32]. The results of several studies have identified CYP24A1 expression in melanoma. CYP24A1 was found to produce bioactive tumor-suppressive vitamin D metabolites (i.e., 20,24(OH)_2_D and 20,25(OH)_2_D) rather than degrading 1,25(OH)_2_D_3_ [33,34,35]. It was also revealed that 20,24(OH)_2_D and 20,25(OH)_2_Dwere more potent vitamin D metabolites as compared to calcitriol and 20(OH)D in inhibiting melanoma growth [33]. The expression of receptors of the 20-hydroxylated vitamin D metabolites—retinoic acid-related orphan receptors α (RORα) and retinoic acid-related orphan receptors γ (RORγ) have been observed to negatively correlate with melanoma progression and positively correlate with melanoma prognosis [34]. Recent evidence suggests that CYP11A1 is also significantly downregulated in numerous types of cancer, including liver, colon, kidney, prostate, lung and uterine corpus endometrial carcinoma [35]. Moreover, CYP11A1 expression is also decreased in prostate cancer bone metastases [36]. RORα and RORγ downregulation have also been related with worse prognosis in patients with lung, breast or liver cancer [37,38,39,40].

Summarizing, the presented relations between the CYP11A1-RORα/γ and progression of tumor suggest that initiation of the alternative vitamin D metabolism pathway may play a crucial role in cancer progression and prognosis.

### 3.3. Mechanism of Vitamin D Action—VDR

The transcription factor VDR is a protein belonging to the conservative ligand-binding nuclear receptor superfamily, which has high affinity to the biologically active form of vitamin D_3_, (1,25(OH)_2_D_3_). VDR is located in over half of the tissues and cell types that build the human body, including bones, kidneys, parathyroid glands, large intestine, skin, brain. Three main domains can be found in the structure of the VDR: the ligand-binding domain (LBD), the DNA binding domain (DBD) and the linker domain (LD). The LBD consists of predominantly nonpolar amino acids forming a pocket, whereby 1,25(OH)_2_D_3_ is bound with high specificity. Bikle, et al. [41] distinguished six domains from A to F in VDR. The first A/B domains constitute the N-terminal region, also known as activation I domain, up to 24 amino acids long. In the C domain, up to 65 amino acids are joined to DNA by two zinc fingers connecting to DNA grooves in the area of vitamin D response elements (VDRE). Next, D domain is a flexible 143-amino acid hinge, followed by an E/F domain made of 195 amino acids binding the ligand and the terminal activation domain—AF2, performing dimerization with and binding to coactivators and corepressors. LBD, composed of 12 helixes, forms a gate mechanism surrounding the ligand, a junction for coactivators and allows for dimerization with retinoid X receptor (RXR) [41,42].

Since VDR has thousands of binding sites in the genome, it influences the transcription of hundreds of genes. It is estimated that there are even over 23,000 VDR binding sites in the human genome, and a large proportion of them are specific to a cell type [41]. In humans, the gene encoding the vitamin D receptor is located on chromosome 12q [43].

In a genomic pathway vitamin D binds to VDR, then undergoes conformational rearrangement enhancing interaction with RXR to form the VDR-RXR heterodimer complex. The resulting VDR-RXR complex translocates to the nucleus, to the promoter containing VDRE and recruits transcriptional coactivators or co-repressors to obtain regulation of mRNA expression of the target genes. The VDRE sequence is highly variable. The role of this complex is remodeling of chromatin and communication with RNA polymerase II to the transcription start site [22,23,27,41,43]. 

There is also a non-genomic mechanism of vitamin D_3_ action, that is based on the interaction with VDR located in the cavities of cell membranes. Two VDR receptors participate in the non-genomic pathway. The first one is the VDR itself located in membrane, it is activated by 1,25(OH)_2_D_3_ analogs, which have a different (6-S cis) configuration than those activating the genomic pathway (6-S trans). The second one is called 1,25(OH)_2_D_3_ membrane-bound rapid-response steroid-binding protein (MARRS). Both receptors reside in the membrane in lipid cave rafts ready to activate ion channels, kinases and phosphatases [27,43,44,45,46]. Vitamin D_3_ activates rapid signal delivery pathways through kinases, phosphatases, or ion channels, which can lead to the alteration of gene expression using cognate promoter elements or regulation of gene expression using VDR as a substrate. The non-genomic mechanism of action is believed to be faster than the genomic one [47].

## 4. Metabolic Phenotype of Vitamin D in Cancer Cells

Physiologically, VDR and CYP27B1 are expressed in numerous tissue in which they govern many functions [48]. However, in many type of cancer cells this VDR and CYP27B1-mediated regulation is disturbed which leads to disorders of vitamin D metabolism and action [24,49,50,51].

### 4.1. CYP27B1

It was observed that CYP27B1 expression is inversely correlated with the progression of tumors of prostate, lung, parathyroid, colon and skin [52,53,54,55,56,57,58], suggesting that local production of 1,25(OH)_2_D_3_ in CYP27B1-expressed tissues could be crucial for cancer prevention. The results of recent study have revealed that pro-inflammatory cytokines including IL-6 and TNF-α decreased CYP27B1 expression in colon cancer cells [59]. Thus, the pro-inflammatory tumor microenvironment is proposed to be a potential factor that reduces CYP27B1 level during tumor progression. The molecular mechanisms responsible for reduced CYP27B1 expression in the process of cancer progression are still not fully understood.

On the contrary, a positive association between reduced CYP27B1 expression and cancer progression was observed in thyroid cancer [60], but inconsistent results were documented for breast [61,62] and renal cancers [63,64]. Additionally, the expression of CYP27B1 in alveolar macrophages from lung cancer patients presented a positive correlation with cancer progression [65]. Notably, pro-inflammatory cytokines including IFN-γ and TNF-α, and Toll-like receptor (TLR) agonists increased CYP27B1 expression in macrophages, monocytes and dendritic cells [62,66]. These observations suggest that the pro-inflammatory tumor microenvironment may contribute to increased CYP27B1 expression in immune cells, which is inconclusive to the outcomes in colon cancer cells [59] mentioned above.

Taken together, CYP27B1 is a biomolecule that may constitute a potential target in cancer therapy. Although, the molecular mechanisms that regulate CYP27B1 expression in particular types of cancer are not fully recognized.

### 4.2. CYP24A1

Taking into consideration that CYP24A1 degrades both calcidiol and calcitriol, its expression might be upregulated by cancer cells and lead to reduced local concentrations of 1,25(OH)_2_D_3_ reported by Albertson et al., who found amplified CYP24A1 in breast cancer [67]. The increased expression of CYP24A1 was demonstrated to be correlated with the advanced stages of prostate, colon, lung and breast cancers, stimulating resistance to vitamin D-mediated therapy [57,61,68,69,70,71,72]. The overexpression of CYP24A1 has been also documented in numerous other types of cancer, including cervical, ovarian, squamous cell and basal cell carcinoma [73,74]. Moreover, CYP24A1 up-expression is related to poor prognosis in colon, lung and esophageal cancer [68,75,76] The oncogenic role of CYP24A1 is supported by results of studies presented that the suppression of CYP24A1 inhibited tumor growth and strengthened antitumorigenic effects of 1,25(OH)_2_D_3_ in breast and lung cancers [77,78,79]. However, opposite data have been also published for prostate cancer [70,80] and a negative correlation between expression of CYP24A1 and tumor progression has been observed in melanoma [81].

It was proposed that increased CYP24A1 expression observed in cancer cells is probably mediated via activation of VDR, because both activity and expression of VDR are downregulated in most types of cancer. Moreover, the overexpression of CYP24A1 in numerous cancer cells may not be a result of normal physiological processes mediated by calcitriol–VDR-dependent mechanisms. Firstly, it has been shown that overexpression of CYP24A1 in breast cancer is related to the amplification of chromosomal locus 20q13.2–20q13.3 comprising the CYP24A1 gene, that has been also found in other types of cancer, including colon malignancies [67,82]. The amplification of CYP24A1 was identified only in malignant, but not benign colon tumors, thus these observations suggest that CYP24A1 overexpression and inactivation of calcitriol may be a key feature of tumor cells [83]. Secondly, epigenetic modifications, namely DNA methylation of the promoter region leads to modification of *CYP24A1* expression in cancer cells. It has been reported that *CYP24A1* expression is negatively correlated with the methylation of the *CYP24A1* promoter in prostate and lung cancer, in vivo and in vitro [75,83] Thirdly, the suppression of DNA methyltransferase (DNMT) or histone deacetylase (HDAC) elevated *CYP24A1* expression in colon and lung cancer. Fourthly, post-transcriptional regulation via microRNAs is related to the *CYP24A1* overexpression in cancer. The expression of *CYP24A1* has been found to be inversely correlated to the expression of miR-125b in breast cancer [84], suggesting that decreased levels of miR-125b may be responsible for *CYP24A1* overexpression in cancer. The results of recent study also showed that the miR-17 to -92 cluster also control *CYP24A1* expression in lung cancer cells [85]. It is also known that the serine/threonine protein kinase casein kinase 2 (CK2) signaling pathway stimulates overexpression of *CYP24A1* in prostate cancers. CK2 is involved in the regulation of *CYP24A1* expression by 1,25(OH)_2_D_3_ and the CK2 inhibitor enhances 1,25(OH)_2_D_3_-mediated antitumor effect [86]. Moreover, *CK2* overexpression has been shown in numerous cancers, including prostate, pancreatic, breast, colon and rectum, lung and bronchus cancer [87]. It should be underlined that *CK2* overexpression was found to be related to poor clinical outcomes [88].

Summarizing, it seems that overexpression of *CYP24A1* may lead to decreased level of calcitriol in cancer.

### 4.3. VDR/RXRα

Progressively reduced expression of *VDR* during dedifferentiation and tumor progression in many types of cancer has been observed. Moreover, a negative correlation between *VDR* expression and tumor malignancy has been shown during the analysis of *VDR* expression levels in normal, benign, and malignant tissues of ovarian, breast, skin and prostate [52,61,89,90,91,92]. Decreased expression of VDR protein has been also observed in urothelial bladder cancer and related with poor prognosis [93]. This evidence suggests that low *VDR* expression may be a potential early diagnostic biomarker for high-risk subjects. Several mechanisms were identified to regulate the expression of *VDR.* Snail1 and Snail2, members of Snail family transcriptional repressor upregulated in may cancers engaged in tumor invasion and metastasis, were revealed to bind to E-boxes in the proximal promoter region of the *VDR* gene leading to recruitment of co-repressors that strength the *VDR* transcription in breast and colon cancer cells [94,95,96]. It has been observed that the expression of H-Ras mutants in rat intestinal epithelial cells and mouse colon as well as the expression of K-Ras mutants in human colon cancer cells suppress calcitriol-mediated activation of VDR activation by inhibiting *VDR* transcription [97]. Moreover, decreased expression of *VDR*, H-Ras and K-Ras mutations in keratinocytes and human prostate epithelial cell lines are related to inhibition of *VDR* transcriptional activity. In turn, suppressed *VDR* transcriptional activity is a result of stimulation of RXR phosphorylation. RXR phosphorylation disturbs the recruitment of co-activator SRC-1 to RXR [98,99].

Epigenetic silencing of *VDR* has been also observed in cancer. The methylation of CpG island in the *VDR* promoter region has been related to decreased VDR expression in breast and colon cancer cells [100,101]. It was also observed that, DNA methyltransferase (DNMT) inhibitor stimulated *VDR* expression and strengthened the anti-proliferative effect of 1,25(OH)_2_D_3_ in breast cancer cells [101]. The engagement of microRNA in the control of VDR expression in cancer has also been proposed. mir-125b was demonstrated to downregulate of *VDR* expression and resulting resistance of melanoma cells to 1,25(OH)_2_D_3_ [102,103].

Taken together, decreased expression of VDR is a distinct feature of cancer cells and is associated with the reduced action of vitamin D.

## 5. Molecular Insight into Anti-Cancer Activity of Vitamin D

### 5.1. Anti-Inflammatory Activity of Vitamin D

Both chronic and acute hyperglycemia trigger increased level of oxidative stress, which in turn contributes to the activation of NF-kB and numerous pro-inflammatory mediators i.e., TNF-α and IL-6. The elevated level of pro-inflammatory cytokines is a key component of low grade inflammation in T2DM subjects [104]. Chronic inflammation extends inflammatory response, leading to progressive destruction and degeneration of tissues by the action of reactive oxygen species (ROS) and cytokines secreted in the site of inflammation. Thereby, chronic inflammation contributes to the initiation of tumorigenesis [105]. Vitamin D exerts anti-inflammatory effects in tumorigenesis via targeting several pathways, including prostaglandin, cyclooxygenase (COX), and mitogen activated protein kinase (MAPK) pathway.

Vitamin D is able to regulate the interaction between immune system and cancer cells resulting in the inhibition of pro-inflammatory cytokines secretion. Co-culture experiments using colon cancer cells and peripheral blood mononuclear cells (PBMCs) showed significant reduction in the secretion of pro-inflammatory cytokines by PBMCs i.e., TNF-α, IL-6 and, IL-10 after vitamin D treatment, supporting the anti-inflammatory properties of vitamin D in tumor microenvironment [106].

**Figure 2:**
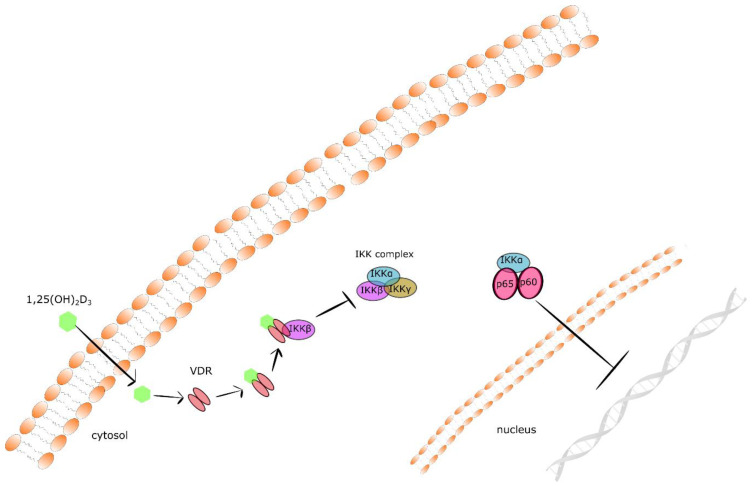
The mechanism of 1,25(OH)_2_D_3_–VDR mediated inhibition of NF-κB activation.

Nuclear factor kappa B (NF-κB) is a well-known master regulator of crosstalk between carcinogenesis and inflammation at multiple levels. Tumorous tissues are characterized by increased NF-κB activity, and the accumulation of pro-inflammatory cytokines creates the so called pro-tumorigenic microenvironment [107]. It has been documented that 1,25(OH)_2_D_3_ suppresses the NFκB signaling pathway. Calcitriol inhibits the phosphorylation of both AKT and its downstream target I kappa Bα (IκBα) via upregulation of thioesterase superfamily member 4 (THEM4) in macrophages. THEM4 is an AKT stimulator protein which upregulation results in the reduction of *NF-κB* and *COX-2* expression [108]. Moreover, 1,25(OH)_2_D_3_ augments the stability of IκBα protein. In fibroblasts, calcitriol augmented the protein stability of IκBα. VDR physically interacts with IκB kinase β (IKKβ) to suppress NF-κB activation. VDR-IKKβ interaction blocks the formation of the IKK complex leading to the inhibition of IKKβ phosphorylation at Ser-177 and abolishing IKK activity to phosphorylate IκBα. Finally, the stabilization of IκBα suppresses the translocation of the p65/p50 complex of NFκB to the nucleus and expression of pro-inflammatory cytokines [109,110]. Together, these data define a novel mechanism of 1,25(OH)_2_D_3_–VDR mediated inhibition of NF-κB activation (presented in Figure 2).

It was proposed that 1,25(OH)_2_D_3_ inhibits prostaglandin pathway engaged in pro-inflammatory responses via the suppression of the cyclooxygenase-2 and prostaglandin receptor EP2 as well as prostaglandin F receptor (*FP*) expression, and degradation of prostaglandins. Additionally, the upregulation of 15-hydroxyprostaglandin-dehydrogenase—NAD^+^-dependent degrading enzyme was observed after exposure to 1,25(OH)_2_D_3_ in prostate cancer cells [111,112]. Reduced mRNA expression of cyclooxygenase-2 and production of prostaglandin E2 have been also documented in 1,25(OH)_2_D_3_-stimulated breast cancer cells [113]. Of note, an inverse correlation between VDR expression and cyclooxygenase-2 expression has been also identified in ovarian cancer tissues and malignant breast cancer cell lines [46,114], supporting the role of 1,25(OH)_2_D_3_-VDR axis in the inhibition of cyclooxygenase expression and prostaglandins production.

P38 MAPK pathway was proposed as both a tumor suppressor and tumor promoter. Despite many studies that provided experimental findings of the antitumorigenic role of p38, many results show also that this kinase promotes cancer development via enhancing migration, survival, resistance to stress and chemotherapeutic agents in tumor cells [45]. 1,25(OH)_2_D_3_ was found to suppress the secretion of pro-inflammatory cytokines i.e., IL-6 via stimulation of MAPK phosphatase-5 expression in both normal prostate epithelial cells and prostate cancer cells. MAPK phosphatase-5 prevents phosphorylation and activation of p38 MAPK [115]. Moreover, 1,25(OH)_2_D_3_ inhibited lipopolysaccharides (LPS)-induced production of IL-6 as well as TNF-α via the activation of MAPK phosphatase-1 in murine macrophages and human monocytes [116].

Taken together, vitamin D shows anti-inflammatory properties, especially by the reduction of pro-inflammatory cytokines expression and regulation of inflammatory signaling pathways.

### 5.2. Antioxidant Properties of Vitamin D

Hyperglycemia, a typical sign of diabetes, leads to elevated production of reactive oxygen species (ROS), and trigger to DNA damage [117]. The major sources of free radicals in people with diabetes are as follows: increased mitochondrial leakage of superoxide anions radical from respiratory chain, glucose autooxidation, oxidative degradation of advanced glycation end-products, activation of sorbitol and hexosamine pathway [13]. The accumulation of free radicals, especially ROS and nitrogen reactive species leads, to the activation of numerous pathways that control apoptosis and differentiation of cells [14,15]. For these reasons, the maintenance of proper function of antioxidant defense systems is a key step in preventing tumor development. It has been also proposed that vitamin D may protect from DNA damage-induced by oxidative stress via the stimulation of antioxidant defenses [118]. Increased oxidative stress-induced DNA damage has been observed in colon epithelial cells of VDR-knockout mice [44]. Additionally, the supplementation of rats with calcitriol significantly decreased level of malondialdehyde—the end product of lipid peroxidation [119]. It has been also found that vitamin D supplementation reduced oxidative DNA damage in human peripheral blood lymphocytes presenting its protective role against oxidative stress-induced DNA damage in humans [120].

The protection against oxidative stress exhibited by vitamin D is related with its molecular mechanism of action that stimulates the expression of numerous enzymes participating in ROS detoxification. It was shown that 1,25(OH)_2_D_3_ stimulated the expression of superoxide dismutase 1 and 2 in prostate epithelial cells and in androgen-sensitive prostate cancer cells [121,122]. Calcitriol also induced the expression of thioredoxin reductase 1 in breast and prostate cancer cells [121,123]. Moreover, 1,25(OH)_2_D_3_ induced expression of glucose-6-phosphate dehydrogenase that is responsible for the production of NADPH for glutathione regeneration in ovarian and prostate cancer cells [124,125]. It has been also shown that NF-E2-related factor-2 (NRF2) increasing antioxidant enzymes’ expression is regulated by vitamin D [126,127]. Vitamin D has been also proposed as a regulator of cellular bioenergetics in the mitochondria in VDR-dependent molecular mechanism. Proposed mechanism is related to the upregulation of numerous molecules engaged in mitochondrial function, especially mitochondrial respiration [128,129]. It is also known that VDR is able to enter the mitochondrion by permeability transition pores [130] and governs its functions [131]. In turn, vitamin D deficiency is related to a decline in the mitochondrial respiration process as a consequence of the decreasing of proteins and nuclear mRNA molecules involved this process [128,129]. Unfortunately, the observed mechanism is still not fully explored [131]. Reduced respiration triggers a drop of mitochondrial bioenergetics leading to changes in oxidative phosphorylation, reduced ATP formation, decreased expression of complex 1 of the electron transport chain, and elevated production of ROS [132]. In turn, increasedROS level decreases the activity of the insulin signaling pathway by lowering of phosphorylation of IRS, GLUT-4 transcription, and alterations of mitochondrial activity [133,134,135]. Observed effects are supported by the findings of the study presenting that 1,25(OH)_2_D_3_/VDR signaling suppresses the process of mitochondrial respiration and differentiation of brown adipose cells [136]. It was also shown that vitamin D in VDR-mediated mechanism protected cells from the excess production of ROS that leads to cell damage [137].

Summarizing, vitamin D exhibits antioxidative properties, especially by the regulation of antioxidants’ genes expression.

### 5.3. DNA Repair Process

Both mitochondrial and nuclear DNA damage are a source of numerous mutations that in turn may trigger malignant transformation [16]. It has been also observed that T2DM is related not only to increased levels of oxidative DNA damage, but also to elevated susceptibility to mutagens and reduced efficiency of DNA repair [17]. Currently, a lot of research into DNA repair disorders in diabetes is conducted. It was revealed that as a result of hyperglycemia the NAD^+^/NADH equilibrium is shifted toward NADH. The relevant level of NAD^+^ is crucial for the activity of poly (adenosine diphosphate-ribose) polymerase (PARP) protein directly involved in the double strand breaks (DSB) repair process. PARP is inhibited by the NHD domain deleted in breast cancer 1 (DBC1) protein, and binding of NAD^+^ to the NHD domain releases PARP and allows DNA-DSB repair [138,139]. Studies performed on podocytes derived from mice models of diabetic kidney disease showed decreased expression of *KAT5*, that is responsible for acetylation of ataxia telangiectasia mutant (ATM), a key protein in DNA-DSB repair. The decreased activation of ATM resulting from diminished expression of *KAT5* disturbs the control of checkpoints connected with cell cycle arrest, DNA repair or apoptosis [140,141]. It was also demonstrated that insulin via the inactivation of glycogen synthase kinase-3 (GSK-3β) led to impaired DNA repair. GSK-3β phosphorylates DNA repair factors such as uracil N-glycosylase (UNG2) participating in single-strand break repair associated with base excision repair (BER) and p53 binding protein 1 (53BP1) involved in repair of DSBs induced during non-homologous end joining (NHEJ) repair [142,143]. Diabetes patients present reduced expression of sirtuin 1 (*SIRT1*) that is responsible for deacetylation of multiple proteins, including transcription factors essential, not only for metabolic machinery, but also for DNA repair. It was found that SIRT1 deacetylated KU70 and FOXO that are recruited to DSBs sites. Thereby, *SIRT1* decreased expression detected in diabetic patients significantly diminishes the efficacy of DNA repair [144,145,146].

Moreover, high concentration of glucose may suppress the expression of DNA repair protein XPD induced by insulin [18]. Thus, both increased levels of DNA damage and decreased efficacy of DNA repair are considered as cancer risk factors. Numerous studies have shown that vitamin D elevates the expression of genes engaged in DNA damage repair including *p53*, proliferating cell nuclear antigen (*PCNA*), and breast cancer 1 (*BRCA1*) in breast cancer cells [123], ATM, recombinant DNA repair protein (*RAD50*) [147], and growth arrest and DNA damage-inducible α (*GADD45α*) in ovarian cancer cells and squamous cell carcinoma (SCC) [148,149]. It has been also observed that vitamin D prevents the degradation of p53-binding protein 1 (53BP1) induced by cysteine proteinase Cathepsin L, that is a lysosomal endopeptidase, in breast cancer cells [150].

To conclude, vitamin D strengthens the DNA repair process by increasing the expression of genes involved in this process.

### 5.4. The Role of Vitamin D in Cell Cycle, Proliferation and Differentiation 

It has been observed that 1,25(OH)_2_D_3_ possesses anti-proliferation and pro-differentiation activities both in normal and malignant cells [151]. The molecular mechanism responsible for the anti-proliferative activity of vitamin D is mediated by growth factor expression, numerous signaling pathways and regulation of the cell cycle. It has been demonstrated that vitamin D upregulates IGFBP3 and the cyclin-dependent kinase (*CDK*) inhibitors, *p21* and *p27*, but downregulates *CDK2*, triggering the reduction of IGF-1- and IGF-2-induced cell proliferation, and thereby cell cycle progression [151]. Moreover, 1,25(OH)_2_D_3_ suppresses the Wnt/β-catenin signaling pathway via the inhibition of the formation of transcription factor 4-β-catenin, (*TCF4-β*)–catenin complexes, or the stimulation of the expression of the Wnt antagonist—Dickkopf-1 (*DKK-1*) [152,153]. Vitamin D-mediated activation of transcription factor, forkhead box O3/4 (*FoxO3/4*), has also been presented. Activated FOXO3/4 regulates the transcription of target genes engaged in cell cycle arrest and anti-proliferation i.e., *GADD45A* through the stimulation of its dephosphorylation and deacetylation in neuroblastoma cells [154]. Vitamin D was also observed to decrease telomerase activity. Moreover, vitamin D induces the expression of transforming growth factor β (*TGFβ*), its receptors, triggering the suppression of breast and colorectal cancer cell growth [155,156].

To sum up, vitamin D may also exert its anticancer activity by suppressing cell proliferation, inducing cell differentiation.

### 5.5. Vitamin D Is Involved in Signaling Pathways Crucial in Tumorgenesis

#### 5.5.1. Transforming Growth Factor β (TGFβ) Signaling Pathway

TGF-β signaling plays an important role in carcinogenesis as both a tumor suppressor and an oncogene. Tumor cells escape antiproliferative effects of TGF-β via mutational inactivation or dysregulation of the expression of components in the signaling pathway. Reduced receptor function and changed ratios of the TGF-β type 1 and type 2 receptors were found in numerous tumor cells. [157]. TGFβ2 is known as a key molecule for the maintenance of tissue homeostasis. Its anti-proliferative properties have been observed in normal epithelial cells and at the early stages of carcinogenesis [158]. The TGFβ-SMAD4 signaling pathway was recognized as responsible for constraining growth and metastatic progression of prostate cancer in PTEN-null mice [159]. The treatment with 1,25(OH)_2_D_3_ or vitamin D analog elevated mRNA expression of *TGFβ2* in MDA-MB-231 and MCF-7 [68], MCF10CA1a [160] as well as primary prostate cancer cells [121]. Interestingly, 1,25(OH)_2_D_3_ and its analog EB1089 induce also expression of *TGFβ1* and *TGFβ* receptors in MCF7 breast cancer cells and 185A1 cells (immortalized mammary epithelial cells) in a mechanism requiring SMAD3 as a co-activator [155]. In turn, 1,25(OH)_2_D_3_ suppressed negative regulators of TGFβ availability, including latent TGFβ binding protein 1 (LTBP1) in OVCAR3 cells [125] andprimary prostate cancer cells [121].

1,25(OH)_2_D_3_–induced upregulation of growth differentiation factor 15 (GDF15) mRNA level has been observed in prostate cancer LNCaP cells [161]. GDF15 was demonstrated as a direct VDR target gene required for 1,25(OH)_2_D_3_–mediated growth inhibition [122]. In prostate cancer PC-3 cells, induced expression of *GDF15* reduced cell proliferation, formation of soft agar clone, and xenograft tumor growth [122,131]. The influence of 1,25(OH)_2_D_3_ on the mRNA expression of other TGFβ family members, including TGFBR1, SMAD6, TGFβ1, was only observed after prolonged treatment in various cell types suggesting that the observed effect may be indirect [162,163].

Bone morphogenic proteins (BMP) are a group of growth factors that belong to the TGFβ superfamily. BMPs play an essential role in the regulation of tissue morphogenesis. In turn, BMP signaling is often disturbed in cancer, including colon cancer [164]. It was also observed 1,25(OH)_2_D_3_ or vitamin D analog regulated mRNA expression of several BMP forms i.e., *BMP6* in primary prostate cancer cells [121], BMP2 and BMP6 in MCF10AT1 cells [160], and TGFβ1 and BMP2A in squamous cell carcinoma lines [162].

#### 5.5.2. Insulin-Like Growth Factor (IGF) Signaling Pathway

Hyperinsulinemia that is associated with diabetes and obesity exerts an effect on cancer development directly, or by IGF and IGF receptors (IGFRs). It has been observed that insulin inhibits IGFBP-1 and thus elevates the free fraction of IGF-1. It is well recognized that aberrant IGF signaling focused on elevated IGF-1R activity is involved in cancer cell proliferation, migration, and invasion [165]. An indirect effect of 1,25(OH)_2_D_3_ on the growth rate of cells, as a result of interfering with the action of growth factors that induce proliferation or increase the secretion of growth factors that stimulate cell differentiation, was also proposed. IGF1-induced cell growth was suppressed by vitamin D analogs in MCF-7 cells. Moreover, observed effect was related to elevated release of IGFBP3 [166]. IGFBP3 is a molecule responsible for limiting the pro-proliferative, anti-apoptotic actions of IGF1 and IGF2 as a result of its binding to them and suppressing their ability to interact with cell surface receptors. Notably, 1,25(OH)_2_D_3_ and vitamin D analogs were found to activate the accumulation of IGFBP3 in primary prostate epithelial cell and prostate cancer cells. In turn, IGFBP3 subsequently suppresses IGF2 action [167,168]. 1,25(OH)_2_D_3_–mediated increased mRNA *expression* of *IGFBP3* was observed in LNCaP prostate cancer cells [161] and RWPE1 cells (immortalized prostate epithelial cell line) [169]. What is more, IGFBP3 was characterized as a critical mediator of 1,25(OH)_2_D_3_–dependent inhibition of LNCaP cell growth [170]. The upregulation of many IGFBP isoforms, including IGFBP3 in prostate tissue have also been observed after a 14-day treatment with the vitamin D analog EB1089 in rats [171].

#### 5.5.3. Wnt-β Catenin Signaling Pathway

The Wnt/β-catenin signaling pathway plays a role in numerous physiological processes, including proliferation, differentiation, apoptosis, migration, invasion and tissue homeostasis. In turn, dysregulation of the Wnt/β-catenin cascade leads to the development and progression of some tumors [172].

Vitamin D is able to arrest the cell growth as a result of disruption of β-catenin function. β-catenin is a terminal mediator of Wnt signaling. In the cytoplasm, β-catenin is found to be associated with adenomatous polyposis coli (APC) tumor suppressor protein. Induction of Wnt signaling triggers the accumulation of β-catenin and its subsequent secretion from APC. Released free β-catenin translocates to the cell nucleus where it binds with the transcription factor TCF4 on DNA strand leading to activation of transcription [173]. In turn, mutations in the APC gene disrupting APC-β-catenin interactions are often present in colon cancer [174]. Vitamin D was reported to block β-catenin-mediated gene transcription in cultured SW480-ADH [175], HT-29 and Caco-2 colon cancer cells [176] by the activation of VDR binding to β-catenin leading to the reduction of the TCF4/β-catenin transcriptional complex formation [175]. It was also observed that injections containing 1,25(OH)_2_D_3_ and 1,25(OH)_2_D_3_ analogs three times a week for 12 weeks significantly decreased polyp number in Apc^Min/+^ mice. Moreover, this effect was related to decreased expression of β-catenin target genes in the small intestine and colon [177]. In HEK293 kidney cells, it was shown that the AF-2 domain of VDR interacts with the C-terminus of β-catenin. [178]. 1,25(OH)_2_D_3_–induced effect can also indirectly govern β-catenin function by elevating the secretion of E-cadherin. E-cadherin is a membrane protein that binds β-catenin and prevents its nuclear accumulation. 1,25(OH)_2_D_3_ treatment was demonstrated to suppress β-catenin-induced gene transcription even in SKBR-3 cells with lack of the E-cadherin gene [178]. Therefore, these data suggest that upregulation of E-cadherin is not the only mechanism for 1,25(OH)_2_D_3_–dependent repression of β-catenin signaling. Reduced levels of nuclear β-catenin, TCF1, CD44, and c-Myc were observed in Apc^Min/+^ mice after 1,25(OH)_2_D_3_ injections [177]. Additionally, 1,25(OH)_2_D_3_ can also exert an effect on the expression of Wnt-signaling regulators such as Wnt activator dickkopf-4 (DKK-4). Vitamin D repressed *DKK-4* [153] and increased expression of the Wnt antagonist dickkopf-1 (*DKK-1*) [179].

### 5.6. Is Vitamin D Involed in Regulation of EMT and Cancer Progression?

Physiologically, epithelial cells maintain apical-basal polarity and contact with adjacent cells via adherent junctions, tight junctions, and desmosomes [180]. After the activation of EMT, tumor epithelial cells lose their cell polarity, cell-cell adhesion and gain migratory and invasive properties, becoming mesenchymal cells [181]. Thus, EMT is a reversible process in which epithelial cells gain mesenchymal morphology and lose intercellular contacts, achieving the ability for invasion and migration [182].

1,25(OH)_2_D_3_ reduced the expression of the mesenchymal marker, vimentin, and elevated the expression of the epithelial marker, E-cadherin. In turn, it triggered to suppression of SKOV-3 cell migration and reduced TGF-β1 induced EMT. Hou et al. have shown that stimulation of SKOV-3 cells by TGF-β1 leads to tumor progression in advanced stages via numerous mechanisms including EMT [182]. Moreover, the results of in vivo and in vitro studies have suggested that 1,25(OH)_2_D_3_ and VDR inhibited the spread of ovarian cancer [183]. It has been also found that 1,25(OH)_2_D_3_ delayed malignant transformation by reducing the expression of β-catenin and elevating the expression of E-cadherin in mouse ovarian surface epithelial cells [184]. The results of animal studies have demonstrated that the exposure of ovarian cancer cells to vitamin D_3_ before the inoculation to immunodeficient mice reduced the potential of the cells to metastasize into lung, liver and bone marrow [185].

DEAD (Asp-Glu-Ala-Asp)-box helicase 4 (DDX4) has been recognized as another molecular target for calcitriol. The exposure to vitamin D decreased the expression of *DDX* which suppressed the invasion of ovarian cancer cells [186]. Interestingly, microarray studies have revealed a number of target genes engaged in tumor growth and progression mediated by 1,25(OH)_2_D_3_. It was also observed that calcitriol downregulates growth-promoting chemokines IL-8, Growth Regulated Protein-β (GRO-β), and GRO-γ [187].

Taken together, vitamin D may inhibit metastasis, especially by decreasing expression of β-cathenin and increasing expression of E-cadherin.

### 5.7. How Does Vitamin D Regulate Apoptosis and Autophagy of Cancer Cells?

It is known that vitamin D induces apoptosis of cancer cells via the downregulation of the anti-apoptotic proteins, Bcl-2 and Bcl-XL, and the upregulation of pro-apoptotic proteins, Bax, Bak, and Bad [188]. Moreover, the stimulation of apoptosis by increased expression of other pro-apoptotic proteins, including death-associated protein (DAP-3), G0-G1 switch 2 (GOS2), Fas-associated death domain (FADD), and caspases has been recently documented [123,163]. Calcitriol also suppresses AKT-mediated anti-apoptotic signaling pathway via upregulation of phosphatase and tensin homolog (PTEN), considered as a tumor suppressor [189]. Vitamin D can also recruit Ca^2+^-dependent apoptotic effectors including Ca^2+^-dependent μ-calpain and Ca^2+^/calpain-dependent caspase-12 [190].

Autophagy plays an important role in both cell survival and apoptosis-independent cell death. An ample evidence suggest that vitamin D is able to switch the mode of autophagy from survival to death in cancer cells [191,192]. Calcitriol-stimulated autophagy was associated with increased expression of beclin-1. The letter interacts with either BCL-2 or PI3K class III, playing a crucial role in the regulation of both autophagy and cell death [193]. Additionally, vitamin D-induced autophagy is a result of the stimulation of the expression of DNA damage inducible transcript 4 (DDIT4) and DNA damage response 1 (REDD1). REDD1 is known as an inhibitor of mechanistic target of rapamycin complex 1 (mTORC1) that suppresses autophagy [194,195].

Summarizing, vitamin D may induce apoptosis of cancer cells and stimulates autophagy.

### 5.8. The Role of Vitamin D in Angiogenesis

Blood vessels are necessary to transport oxygen and nutrients for growth and metastasis of cancer cells. Growing cancerous tissue secretes numerous proteins, including EGF, estrogen, basic and acidic FGF, IL-8, prostaglandin E1 and E2, TNF-α, and VEGF. These molecules may activate endothelial cell growth and motility when the production of anti-angiogenic factors is decreased [196].

1,25(OH)_2_D_3_ has been found to have an antiangiogenic effect by modulating the hypoxia-inducible factor 1 (HIF-1) pathway in human cancer cells. Hypoxia is the main trigger of angiogenesis in tumors. HIF-1 is a key transcription factor regulating angiogenesis. It has been documented that 1,25(OH)_2_D_3_ decreases the expression of the HIF-1α subunit, VEGF and inhibits cancer cell proliferation under hypoxic conditions [197]. The antiangiogenic effect of 1,25(OH)_2_D_3_ on tumor endothelial cells may also be VDR mediated. In VDR knockout animals, elevated vascular volume and reduced number of pericytes responsible for regulation of the proliferation of endothelial cells was observed [198].

## 6. What Do We Know So Far about Vitamin D in Cancer Prevention and Prognosis from Clinical Trials?

The results of clinical trials evaluating the relationship between vitamin D and cancer risk are inconclusive. It seems that multiple factors such as age and sex of participants, serum level of 25(OH)D before cancer diagnosis, the dose and type of vitamin D, the duration of supplementation, type and grade of cancer and many others, affect this relationship. A systematic review performed by Bjelacovic et al. collected the results of eighteen randomized clinical trials that assessed cancer risk after vitamin D supplementation versus placebo or no intervention. To the analysis were included studies that compared different doses of vitamin D intake, administered by any route and various duration of supplementation. The participants took the vitamin in the form of a supplement as vitamin D_3_ (cholecalciferol) or vitamin D_2_ (ergocalciferol), and in the active form as 1α-hydroxyvitamin D or 1,25(OH)_2_D_3_ (calcitriol). Fourteen studies tested vitamin D3, one study examined the effects of vitamin D2, and in the remaining three studies calcitriol supplementation. All samples for the study were sourced from high-income countries. The study was attended by participants from the age bracket of 47 to 97 years, and an average of 81% were women. Vitamin D was given for approximately 6 years. The systematic review showed no effect of vitamin D on overall cancer risk (relative risk (RR) = 1.00; 95% CI 0.94–1.06). Site-specific cancers, including lung cancer, breast cancer, colorectal cancer, pancreatic cancer, and prostate cancer, were also tested, with no evidence of the prevention of vitamin D against cancer occurrence (one test each). Therefore, the findings from the systematic review by Bjelakovic et al., do not support anticancer action of vitamin D. However, vitamin D was found to reduce cancer mortality and all-cause mortality [199]. We also searched for meta-analyses and systematic reviews of observational and interventional trials that assessed the relationship between serum concentration of 25(OH)D and the risk of site-specific cancer, including: breast, colorectal, bladder prostate, lung, ovary, pancreas and kidney as presented in Table 1. Gandini et al. [200] performed a meta-analysis of observational studies that determined the association between vitamin D serum concentrations and the incidence of breast, colorectal and prostate cancer. An inverse relationship was found between serum 25-hydroxyvitamin D levels and colorectal cancer. In the analyzed studies, no correlation was found between the level of vitamin and the occurrence of breast and prostate cancer. In turn, a systematic review of eight studies by Shao et al. [201] revealed that higher 25(OH)D blood levels are associated with a lower risk of breast cancer. However, the study did not consider whether vitamin D concentrations were measured before or after the diagnosis of the cancer. Lee et al. [202] found no significant relationship between serum 25(OH)D levels and the overall risk of colorectal cancer. In the research by Ma et al. [203], nine studies were analyzed, one study involving participants from Asia, six studies from the United States and two studies from Europe, however, the results were inconsistent, In turn, Chandler et al. [204] in 2015, conducted a risk assessment of colorectal cancer in women. The results confirmed a possible relationship between serum 25(OH)D concentration and the risk of colon cancer. The strongest reduction in morbidity was observed for 25(OH)D levels higher than 29 ng/mL. Weinstein et al. [205], Xu et al. [206] and Zhang et al. [207] confirmed that patients with higher vitamin D levels may have a reduced risk of cancer and a better prognosis. In turn, the incidence of bladder cancer was determined by Zhang et al. [208] based on the results from observational studies, they suggest that low serum vitamin D levels are associated with an increased risk of bladder cancer. While the intervention studies conducted by Zhao et al. [209] provided more information that patients with serum 25-hydroxyvitamin D concentration > 75 nmol/L had the lowest risk of bladder cancer compared to patients with 25-hydroxyvitamin D < 25 nmol/L and moderate vitamin D deficiency (25–37.5 nmol/L). However, it should be highlighted that a vitamin D level that is too high is thought to increase cancer risk. Xu et al. [210] revealed a significant 17% increased risk of prostate cancer in men with higher 25(OH)D levels. In the study by Schenk et al. [211], men 55 years of age or older with biopsy proven prostate cancer were studied. Tumors were assessed using the Gleason scale. It was found that a higher serum 25(OH)D concentration might slightly increase the risk of Gleason 2–6 disease and significantly reduce the risk of prostate cancer on the Gleason 8–10 scale. In the case of pancreatic cancer, Stolzenberg-Solomon et al. [212] noted that, compared with concentrations of 50.0–75.0 nmol/L of vitamin D, concentrations of 100 nmol/L or higher were associated with an elevated risk of pancreatic cancer. Thus, these observations provide rationale for careful recommendations of vitamin D supplementation to healthy people with physiological 25(OH)D serum concentration for the prevention of cancer. On the other hand, Wolpin et al. [213] suggest that higher 25(OH)D serum levels were associated with a lower risk of pancreatic cancer and low 25(OH)D levels might predispose to the development of pancreatic cancer. Interestingly, this study did not report an increased risk of pancreatic cancer among people with 25(OH)D > 100 nmol/L. Li et al. conducted a case-control study in the Chinese Han population in which they found the protective effect of higher 25(OH)D levels against renal cell carcinoma [214]. To sum up, the available results of clinical trials, mainly observational, do not allow one to unambiguously state that supplementation with vitamin D reduces the risk of cancer. There is a pressing need for, randomized interventional clinical trials to clarify the amount of vitamin D and the duration of supplementation needed to obtain a protective benefit against cancer. 

Considering the molecular action and metabolism of vitamin D, VDR expression in cancer cells, it is very interesting how the vitamin affects cancer prognosis. To address this problem, we searched for studies aimed at determining the effect of vitamin D level at the time of cancer diagnosis or its supplementation after diagnosis or surgery on a 5-year overall survival (OS) and progression-free survival (PFS) in patients suffering from cancer. Vaughan-Shaw et al. performed a stratified analysis of 64 clinical trials involving patients suffering from various cancers that revealed the improvement of overall survival, and survival without progression was associated with 25(OH)D ⩾20 ng/mL [222]. However, the beneficial effect of a high vitamin D level was not always found in site specific cancers as displayed in Table 2 and Table 3.

Yuan et al. evaluated the relationship between vitamin D level, and progression and survival of patients with advanced or metastatic colorectal cancer. This prospective study involved 1041 patients with previously untreated colorectal cancer, who participated in phase III clinical trial testing first-line chemotherapy and biological therapy. Participants were categorized into five groups according to 25(OH)D level: 1: 2.2–10.8 ng/mL; 2: 10.9–15.4 ng/mL; 3: 15.4–19.2 ng/mL; 4: 19.3–24.0 ng/mL; 5: 24.1–72.7 ng/mL. Only 6% of patients had a vitamin D sufficient level (≥30 ng/mL), 31% had vitamin D insufficiency (vitamin D 20–<30 ng/mL), and 63% of patients had vitamin D deficiency (<20 ng/mL). Participants with 25(OH)D concentration > 24.1 ng/mL had improved OS and PFS than those with 25(OH)D concentration < 10.8 ng/mL [223]. Similarly, the results of analysis performed by Yang et al. also suggested that the level of 25(OH)D higher than 29.9 ng/mL was related to favorable OS in patients with stage I–III colorectal cancer [224]. A retrospective analysis by Wesa et al. [225] revealed that stage IV colorectal cancer patients who had serum 25(OH)D levels ≥ 30 ng/mL presented better OS than those with 25(OH)D levels < 30 ng/mL.

Urashima et al. [226] conducted a randomized, double-blinded, placebo controlled study (AMATERASU) in which, patients aged 30 to 90 years old with tumors of the gastrointestinal tract (from the esophagus to the rectum) in various stages of development were supplemented with 2000 IU of vitamin D orally from the first day of the postoperative visit until the end of the study. They found that vitamin D supplementation did not significantly improve the 5-year relapse-free survival among patients with gastrointestinal cancer. Yonaga et al. performed a *post hoc* analysis of the AMATERAS study, namely participants were divided based on histopathological characteristic into the following subgroups: squamous cell carcinoma; adenocarcinoma well differentiated, moderately differentiated or poorly differentiated. They found that postoperative oral vitamin D supplementation improved OS and PFS in the subgroup of patients with poorly differentiated gastrointestinal adenocarcinoma, but not in the remaining subgroups [227]. Interestingly, a *post hoc* analysis of AMATERAS study conducted by Akutsu et al. in which participants were stratified based on p53 protein, vitamin D receptor and Ki-67 expression levels in tumor samples demonstrated that daily 2000 IU vitamin D supplementation significantly improved PFS and a non-significant 10% longer 5-year OS in the subgroup of patients with p53 positive tumors [228].

Interesting findings concerning pancreatic cancer, that is characterized by high resistance to treatment and short patients’ survival typically no longer than a year, were performed by Van Loon et al. [229] and Zhang et al. [230]. Van Loon et al. found among patients suffering from advanced pancreatic cancer at the time of diagnosis, 44.5% of patients had vitamin D deficiency (<20 ng/mL), and 22.5% had vitamin D insufficiency (30–20 ng/mL). The baseline vitamin D level was not associated with OS or PFS. In turn, the meta-analysis of twelve studies by Zhang et al. demonstrated that high serum 25(OH)D level was related to decreased pancreatic cancer mortality and improved OS [230]. 

The results of several studies suggest that low vitamin D level is associated with poor prognosis of different types of lymphoma. Kelly et al. reported that vitamin D may be an important, modifiable factor related to survival in people with follicular lymphoma prior to the implementation of specialist treatment [232]. Although, not statistically significant Tracy et al. [233] found a trend toward vitamin D deficiency (<20 ng/mL) and worse OS and PFS in patients suffering from follicular lymphoma. Additionally, Drake et al. noted worse PFS and OS in DLBCL (diffuse large B-cell lymphoma) and T-cell lymphoma in patients with 25(OH)D level < 25 ng/mL [234]. 

Very important observations were made by Kanstrup et al. who found that women suffering from primary invasive breast cancer with 25(OH)D level ≤ 52 nmol/L had worse PFS and OS compared to women with 25(OH)D level between 76–99 nmol/L. Interestingly, women with breast cancer with 25(OH)D level ≥ 99 nmol/L also had worse PFS and OS [237]. Of note, Xu et al. showed that the level of VDR protein expression in neoplastic cells in patients with breast cancer was related to OS, therefore, they recommned individualized vitamin D intake to assess breast cancer prognosis [238]. In case of lung cancer, Huang et al. conducted a meta-analysis of eight cohort studies to evaluate the correlation between serum 25(OH)D level and OS. The obtained results suggested a correlation between low serum 25(OH)D concentration and poor OS of patients with lung cancer [235].

Few studies have examined how vitamin D supplementation after surgery affects cancer prognosis. Akiba et al. conducted a randomized, double-blinded, placebo-controlled study with 1200 IU vitamin D supplementation per day for 1 year, after surgery, in patients suffering from lung cancer. RFS and OS were compared between the groups with lower < 20 ng/mL and higher ≥ 20 ng/mL 25(OH)D levels. Among patients suffering from early adenocarcinoma, those with low 25(OH)D levels had significantly worse OS than patients with higher 25(OH)D. They did not find any significant improvement of PFS and OS in lung cancer patients in the overall study population. including advanced stages and squamous cell carcinoma or large cell carcinoma [236]. Attia et al. conducted a study in which a standard treatment of prostate cancer (docetaxel), doxercalciferol (vitamin D analogue) once weekly, was added in phase I and II clinical trials. However, they did not report the improvement in survival of patients with prostate cancer [231]. An important problem was raised by Markotic et al., who measured serum 25(OH)D level in patients with colorectal cancer before and after surgery, due to the fact that surgical treatment has a great influence on the bowel absorption of vitamin D. Participants were classified to sufficient vitamin D group (25(OH)D > 20 ng/mL) and insufficient vitamin D group (25(OH)D < 20 ng/mL). They revealed that the serum levels of 25(OH)D higher than 20 ng/mL were associated with better OS only in the postoperative period, but not in the preoperative period [20,239] as presented in Table 3.

## 7. What Do We Know from Clinical Trials about Vitamin D Action in Diabetes?

Systematic review performed by Pittas et al. showed the association between low vitamin D level and type 2 diabetes risk [240]. However, the results of the systematic review should be interpreted cautiously because of the limited number of available evidence. The majority of included observational studies were cross-sectional, biased due to uneven distribution of confounding factors, while the interventional studies were short-term, included small number of participants, used vitamin D_3_ or D_2_ or its analogues, or contained *post hoc* analyzes. Evidence from studies with vitamin D suggest that its supplementation may prevent from type 2 diabetes only in high-risk (i.e., glucose intolerant) patients [240]. The effect of vitamin D supplementation on the risk of convertion of prediabetes to type 2 diabetes was evaluated in the study performed by Niroomand et al. [241]. It included patients with prediabetes and hypovitaminosis D who were supplemented with 50,000 IU of vitamin D or placebo. It showed that vitamin D improved insulin sensitivity and reduced the risk of prediabetes to diabetes progression [241]. However, serious limitations such as small number of participants (162 cases) and short duration should be recognized. In turn, the interventional study, the Diabetes Prevention with active Vitamin D (DPVD), conducted by Kawahara et al. aimed to assess the effect of eldecalcitol, an active vitamin D analogue, on the incidence of type 2 diabetes in subjects with pre-diabetes among Japanease men and women over 30 years of age. They reported 121 diabetes cases after a median follow-up of 2.8 years, including 57 in the eldecalciol group and 64 in the control group [242].

The results of our study demonstrated that three-month supplementation with vitamin D of the elderly with metabolic disorders significantly increased HDL level and decreased HOMA-IR. and TG/HDL ratio. Furthermore, we observed non-significant reduction of HbA1c (0.5%) in subgroup with T2DM after vitamin D supplementation [243]. Similarly, Upreti et al. noted that six-month supplementation with vitamin D of T2DM patients triggered decrease of HbA1c [244]. In turn, Mirrhosseini et al. observed that vitamin D reduced fasting plasma glucose (FPG), HbA1c, and HOMA-IR leading to improvement of glycemic control [245]. The study carried out by El Hajj et al. shown that vitamin D contributes to markedly decrease of FPG, HOMA-IR, TC and LDL [246]. In turn, co-supplementation of vitamin D with calcium reduced HbA1c, serum insulin level, LDL, HOMA-IR, and TC/HDL. Moreover, elevated quantitative insulin sensitivity check index (QUICKI) and HDL were also detected [247]. Barzegardi et al. reported reduced serum levels of LDL, TG, and TC in patients with diabetic nephropathy after vitamin D supplementation [248]. Summarizing, the results of presented studies indicate that vitamin D improves insulin resistance resulting in better metabolic control of diabetes.

Increased concentrations of hs-CRP and pro-inflammatory cytokines i.e., TNF-α and IL-6 has been observed in patients with low level of vitamin D [249,250,251,252,253,254]. The results of some studies have revealed that supplementation with vitamin D reduced level of circulating pro-inflammatory biomarkers (TNF-α. IL-6) in T2DM patients [110,255,256]. However,.Beilfuss et al. have not observed the influence of vitamin D on TNF-α in obese subjects [257,258,259,260]. The results of meta-analysis carried out by Yu et al. did not find any significant evidence that supplementation with vitamin D changed levels of TNF-α and IL-6 in T2DM patients [261].

## 8. Vitamin D in Cancer Prevention among Diabetes Patients

Diabetes and cancer are common chronic diseases, which frequently co-exist. Grow-ing body of evidence shows that patients with diabetes are more susceptible to the development of different cancers. The causative factors of this increased coexistence are not fully recognized. It is believed that shared pathophysiology and/or environmental risk factors may be responsible for the excess cancer risk in diabetic patients. Numerous evidence which includes epidemiological, experimental and clinical studies suggests that both cancer development and T2DM development are increased in subjects with inadequate vitamin D levels. Therefore, it can be assumed that in diabetic patients with vitamin D deficiency, the risk of cancer development will be accumulated. Taking into account the pleiotropic effect of vitamin D, especially engagement in insulin synthesis and secretion, immune response, regulation of calcium intracellular level, and response to insulin, its deficiency contributes to the intensification of typical symptoms of diabetes, such as insulin resistance, hyperinsulinism, hyperglycemia and low grade chronic inflammation. Thus, the altogether disorders accompanying diabetes create a microenvironment leading to the development of cancer, and vitamin D deficiency exacerbates their intensity. Most of the results of clinical trials involving patients suffering from T2DM show that supplementation with vitamin D improves the level of metabolic parameters associated with insulin resistance, hyperinsulinemia, hyperglycemia and low grade chronic inflammation. However, there are no clinical trials evaluating the impact of vitamin D supplementation on cancer risk among patients suffering from diabetes. Only, in the study by Wang et al. that aimed to determine the association between serum 25(OH)D concentrations and can-cer-specific mortality in 1188 older post-menopausal women, we found that diabetes did not significantly increase cancer mortality with a vitamin D cutoff of 64 nmol/L (25.6 ng/mL) [262]. Thus, there is a pressing need for randomized clinical trials to clarify whether vitamin D deficiency may be another factor responsible for increased risk of cancer in T2DM patients, and whether the use of the vitamin by patients with diabetes may decrease cancer risk.

## 9. Conclusions

**Figure 3:**
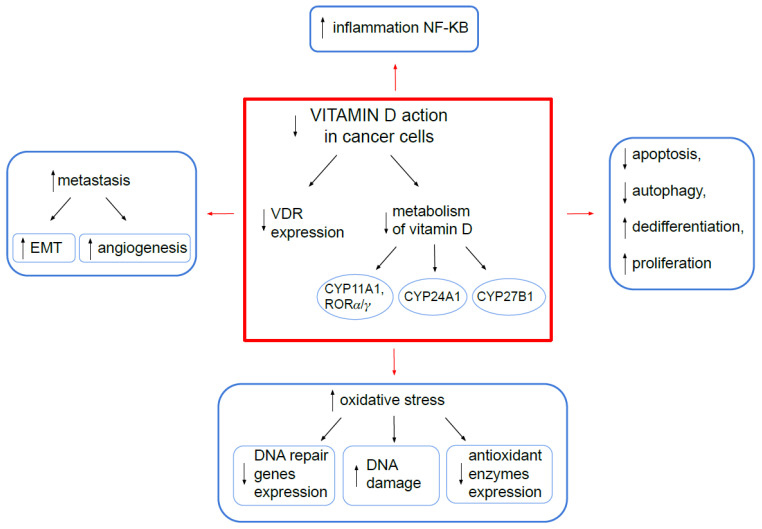
The potential mechanisms that link vitamin D deficiency and increased risk of cancer in T2DM.

Although poorly explored, it seems that vitamin D deficiency may be one of the crucial factors responsible for increased cancer risk among T2DM patients. On the one hand, cancer cells present disturbances in expression of VDR and CYP27B1 that lead to disorders of vitamin D metabolism and action. On the other hand, vitamin D deficiency impairs multiple cancer-related cellular processes such as DNA repair process, apoptosis, autophagy, and signaling pathways involved in tumorigenesis i.e., TGFβ, IGF and Wnt-β-Cathenin. Of note, recognized molecular mechanisms of vitamin D action prevent or alleviate T2DM-related disorders such as insulin resistance, hyperinsulinemia, hyperglycemia, oxidative stress, and inflammation that are well recognized players in tumorigenesis. Figure 3 summarizes the association between vitamin D deficiency and related disturbances in numerous cellular processes connected with T2DM- cancer relationship.

## Figures and Tables

**Table 1:** Studies of blood 25(OH)D concentrations and cancer risk.

First Author/Year of Publication	Type of Study	No. of Studies/Cases	Relative Risk (RR)	95% CI	
BREAST CANCER					
Gandini, 2011	Meta-analysis of observational studies	10 studies/6175 cases	0.89	0.81–0.98	
	Case-control study (retrospective)	3030 cases	0.83	0.79–0.87	
	Prospective study	3145 cases	0.97	0.92–1.03	
Shao, 2012 [201]	Meta-analysis of observational case-control studies	8 studies	0.55	0.38–0.80	
Wang, 2013 [215]	Meta-analysis of prospective studies	14 studies/9110 cases	0.85	0.75–0.95	
Kim, 2014 [216]	Meta-analysis of prospective studies	14 studies/9526 cases	0.92	0.83–1.02	
Kim, 2014 [217]	Nested case-control of observational study	707 cases	0.43	0.23–0.80	
Skaaby, 2014 [218]	Cohort study	159 cases	1.02	0.96–1.09	
COLORECTAL CANCER					
Lee, 2011 [202]	Meta-analysis of prospective studies	8 studies	0.66	0.54–0.81	
Gandini, 2011 [200]	Meta-analysis of observational studies	9 studies/2630 cases	0.85	0.79–0.91	
Ma, 2011 [203]	Meta-analysis of prospective studies	9 studies/2767 cases	0.67	0.54–0.80	
Chandler, 2015 [204]	Nested case-control of observational study	274 cases	0.45	0.25–0.81	
Weinstein, 2015 [205]	Nested case-control study	476 cases	0.60	0.38–0.94	
Xu, 2018 [206]	Meta-analysis of observational studies	11 studies/7367 cases	0.67	0.56–0.80	
Zhang, 2019 [207]	Meta-analysis of observational studies	8 studies/2916 cases	0.75	0.58–0.97	
PROSTATE CANCER					
Gandini, 2011 [200]	Meta-analysis of observational studies	11 studies/3956 cases	0.99	0.95–1.03	
Xu, 2014 [210]	Meta-analysis of observational studies	21 studies/11,941 cases	1.17	1.05–1.30	
Schenk, 2014 [211]	Nested case-control study	1695 cases	1.10	0.90–1.35	
	Gleason 2–6 score		1.21	0.97–1.52	
	Gleason 7 score		1.09	0.78–1.52	
	Gleason 8–10 score		0.55	0.32–0.94	
LUNG CANCER					
Zhang, 2015 [208]	Meta-analysis of observational studies	12 studies/288,778 participants	0.84	0.78–0.90	
Chen, 2015 [219]	Meta-analysis of observational studies	13 studies/2227 cases	0.95	0.91–0.99	
OVARY CANCER					
Yin, 2011 [220]	Meta-analysis of observational studies	10 studies	0.83	0.63–1.08	
FIRST AUTHOR/YEAR OF PUBLICATION	TYPE OF STUDY	NO. OF STUDIES/CASES	COMPARISION	RELATIVE RISK (RR)	95% CI
BLADDER CANCER					
Zhang, 2015 [221]	Meta-analysis of observational studies	7 studies/62,141 participants		1.34	1.17–1.53
Zhao, 2016 [209]	Meta-analysis of interventional studies	7 studies/2509 cases	>75 nmol/L vs. <25 nmol/L 25(OH)D	0.68	0.52–0.87
			>75 nmol/L vs. 25–37.5 nmol/L 25(OH)D	0.65	0.49–0.86
			>75 nmol/L vs. 37.5–50 nmol/L 25(OH)D	0.61	0.47–0.80
			>75 nmol/L vs. 50–75 nmol/L 25(OH)D	0.65	0.48–0.85
PANCREAS CANCER					
Stolzenberg-Solomon, 2010 [212]	Meta-analysis of nested case-control studies	8 studies/952 cases	>100 nmol/L vs. 50–75 nmol/L 25(OH)D	2.12	1.23–3.64
Wolpin, 2012 [213]	Meta-analysis of nested case-control studies	5 studies/451 cases	<50 nmol/L vs. 50–75 nmol/L 25(OH)D	0.75	0.58–0.98
			≥75 nmol/L 25(OH)D	0.71	0.52–0.97
KIDNEY CANCER					
Li, 2019 [214]	Case-control study	135 cases	20–30 ng/mL 25(OH)D	0.50	0.29–0.88
			≥30 ng/mL vs. <20 ng/mL 25(OH)D	0.30	0.13–0.72

**Table 2:** The effect of 25 (OH) D on the OS and PFS in various types of cancer.

			OS (Overall Survival)		PFS (Progression-Free Survival)	
First Author/Year of Publication	No. of Studies/Cases	Median Follow-Up TIME	Relative Risk (RR)	95% CI	Relative Risk (RR)	95% CI
COLORECTAL CANCER						
Wesa, 2016 [225]	250 cases	2 years	0.61	0.38–0.98	-	-
Yang, 2017 [224]	206 cases	45 months	0.442	0.238–0.819	-	-
Yuan, 2019 [223]	1041 cases	31.2 moths	0.66	0.53–0.83	0.81	0.66–1.00
DIGESTIVE TRACT CANCER						
Urashima, 2019 [226]	439 cases	3.5 years	0.76	0.5–1.14	0.95	0.57–1.57
Yonaga, 2019 [227]	-	-	0.25	0.07–0.94	0.25	0.08–0.78
Akutsu, 2020 [228]	372 cases	-	0.66	0.34–1.27	0.52	0.31–0.88
PROSTATE CANCER						
Attia, 2017 [231]	70 cases	17.8 months	17.8	14.9–23.6	6.17	4.20–10.7
FOLLICULAR LYMPHOMA						
Kelly, 2015 [232]	777 cases	5.4 years	4.16	1.66–10.44	1.97	1.10–3.53
Tracy, 2017 [233]	642 cases	59 months	2.35	1.37–4.02	2.05	1.18–3.54
NON-HODGKIN’S LYMPHOMA						
Drake, 2010 [234]	983 cases	34.8 months	Large B-cell lymphoma (DLBCL)			
			1.99	1.27–3.13	1.41	0.98–2.04
			T-cell lymphoma			
			2.38	1.04–5.41	1.94	1.04–3.61
PANCREATIC CANCER						
Van Loon, 2014 [229]	256 cases	5.8 months	1.00	0.99–1.01	1.00	0.99–1.01
Zhang, 2017 [230]	12 studies	-	0.6	0.45–0.97	1.06	0.84–1.33
LUNG CANCER						
Huang, 2017 [235]	8 studies/2166 cases	-	1.30	1.08–1.55	-	-
Akiba. 2018 [236]	155 cases	3.3 years	1.22	0.54–2.79	1.15	0.64–2.05
OVERALL CANCER						
Vaughan-Shaw. 2017 [222]	64 studies/44 155 cases	-	0.74	0.66–0.82	0.84	0.77–0.91
BREAST CANCER						
Kanstrup. 2019 [237]	2510 women	5.59 years	25(OH)D ≤ 52 nmol/L			
			1.55	1.06–2.25	1.63	1.21–2.19
			25(OH)D ≥ 99 nmol/L			
			1.20	0.88–1.66	1.37	1.02–1.83
Xu. 2020 [238]	8 studies/2503 cases	-	0.41	0.18–0.95	1.14	0.87–1.50

**Table 3:** The effect of preoperative and postoperative 25 (OH) D levels on the OS in patients with colorectal cancer.

First Author/Year of Publication	No. of Studies/Cases	Relative Risk (RR)	95% CI
COLORECTAL CANCER			
Markotic. 2019 [239]	515 (286 pre-operatively and 229 post-operatively)	pre-operatively	
		0.53	0.33–0.84
		post-operatively	
		1.13	0.77–1.65
